# Economic Analysis of Inequality in Preventive Health Check-Ups Uptake in Saudi Arabia

**DOI:** 10.3389/fpubh.2021.745356

**Published:** 2021-09-17

**Authors:** Mohammed Khaled Al-Hanawi, Gowokani Chijere Chirwa

**Affiliations:** ^1^Department of Health Services and Hospital Administration, Faculty of Economics and Administration, King Abdulaziz University, Jeddah, Saudi Arabia; ^2^Health Economics Research Group, King Abdulaziz University, Jeddah, Saudi Arabia; ^3^Economics Department, Chancellor College, University of Malawi, Zomba, Malawi

**Keywords:** check-ups, healthcare, inequality, Saudi Arabia, preventive

## Abstract

**Background:** Undertaking preventive health check-ups has proven to be an important strategy in the fight against several diseases. However, various socioeconomic circumstances may hinder participating in such an important health exercise for many people. With the growth in the burden of non-communicable diseases in Saudi Arabia, it is thus essential that people take an active role in undertaking preventive health check-ups. However, the extent to which this behavior is determined by inequalities in socioeconomic circumstances remains not well-documented. The aim of this study was to examine the socioeconomic inequalities in undertaking preventive health check-ups in Saudi Arabia, using a national survey with a sample of 11,528 respondents.

**Methods:** Data from the Saudi Family Health Survey conducted in 2018 by the General Authority for Statistics were used for the analysis of this study. Univariate, bivariate, and multivariate logistic regression analyses were employed to examine the socioeconomic factors associated with undertaking preventive health check-ups. Concentration indices were calculated, and associated concentration curves were used to assess the socioeconomic inequalities in preventive health check-ups uptake. Moreover, decomposition analysis was performed to examine the extent to which the socioeconomic variables affect uptake of preventive health check-ups.

**Results:** The results reveal that being older adults, more educated, insured, and married increase the probability of undertaking preventive health check-ups. Regarding socioeconomic inequalities, preventive health check-ups uptake was concentrated among the wealthier (concentration index: 0.0831; *P* < 0.001). However, some differences were observed in terms of socioeconomic inequality across the regions. Decomposition of the Erreygers index supported the analysis of the determinants and suggested that income, and education were the primary drivers of the associated inequality.

**Conclusions:** These results suggest that the government of Saudi Arabia should develop intervention programs and strategies that promote the uptake of health check-ups among the vulnerable group to reduce inequalities. Of particular importance is the need for more health-related education among the poor and those with lower education in order to raise their awareness on the benefits and advantages of conducting health examinations.

## Introduction

Healthcare services in the Kingdom of Saudi Arabia (KSA) are provided through the public sector, including the Ministry of Health (MOH) and other government sectors, as well as through the private sector. The public healthcare sector is operated, financed, and managed by the government. The bulk of healthcare service provision in the KSA is undertaken by the public healthcare sector through the MOH, which is funded by government revenue through the annual allocated budget (1). The MOH currently provides healthcare services to all Saudi citizens free of charge at the point of use, covering 2,390 primary healthcare centres and 284 public hospitals (2). Government sectors outside the MOH provide healthcare to a defined population, usually consisting of employees and dependents of the respective ministries and public sector organizations. By contrast, the private, “for-profit” healthcare sector is operated, financed, and managed by either individuals or companies (3). The private sector provides healthcare services based on a fee for service, paid for out of pocket by the patients or by private health insurance plans. Approximately, 62.4% of the total health expenditure is from government while the rest from other means including out-of-pocket spending and voluntary health insurance contributions (4).

The sustainable development goals (SDGs) have emphasized reducing inequalities in health outcomes and access (5). To maintain a good health status, it is important to undertake regular medical check-ups as opposed to waiting until symptoms or diseases manifest (6). Primary and secondary preventive health services that include public health check-ups are extremely important and positively influence health behavior (7). Evidence showed that health check-up services can promote health, reduce inpatient and outpatient service use and expenditures (8–11). While several countries around the world have developed national guidelines for implementing health promotion programs and preventive health check-ups programs, some countries lack health promotion and preventive health initiatives (12).

Measuring the uptake of preventive health check-ups is important, as it provides a signal as to how many people may be aware of their health and can help to detect early chronic conditions, among others (13). Nevertheless, undertaken preventive health check-ups has been influenced by a variety of sociodemographic and economic factors. Previous studies reported socioeconomic inequalities in undertaking health check-ups (14, 15). Various socioeconomic inequalities may contribute to a difference in the uptake of preventive health check-ups (16).

In the KSA, only a handful of studies have explored certain aspects of preventive healthcare in the KSA. One study showed that people living in rural areas of Riyadh faced considerable barriers to actively participating in their own healthcare as opposed to urban residents (17). Another study investigated the effect of health insurance on preventive healthcare, which established that the insured are more likely to undertake some medical check-ups (18). Interestingly, studies exploring and decomposing inequalities in preventive healthcare are scarce. Although some studies have explored socioeconomic inequalities in healthcare, they have largely focused on specific conditions such as diabetes (19) and breast cancer (20).

Given that healthcare services are provided largely free of charge in the KSA, there is little evidence available to examine if people are actively participating in their own preventive health by undertaking health check-ups. With an emerging chronic disease burden in the KSA (19), and the increase in the life expectancy associated with the increase of the elderly population (21, 22), studying the uptake of preventive healthcare behavior may be of significance. Hence, the aim of this study was to measure the extent of socioeconomic inequalities in undertaking health check-ups in the KSA. Furthermore, the observed socioeconomic inequality in undertaking health check-ups was decomposed to identify the main determinates contribute to the observed inequality.

To the best of our knowledge, no study has specifically analyzed socioeconomic inequalities in undertaking preventive health check-ups in the KSA. Specifically, no study has used concentration indices to examine the inequality dimensions of undertaking preventive periodic health check-ups. Therefore, this study aimed to fill this gap in the literature by measuring socioeconomic inequalities in undertaking preventive health check-ups in the KSA using a rich dataset with national representativeness. In particular, this study contributes to the existing literature by quantifying the extent of income- and education-related inequalities in preventive healthcare in Saudi Arabia.

## Materials and Methods

### Data Source and Sample

This study used data obtained from the Saudi Family Health Survey (FHS), which was conducted in 2018 by the General Authority for Statistics (GaStat) (23). The FHS is classified as a family survey falling under the categories of education and health statistics. The FHS was a collaboration among the GaStat, MOH, and Saudi Health Council, in addition to the academic and private sectors. The FHS collects information by visiting a representative sample of the population across all administrative regions in the KSA.

The survey contains several questions that obtain information relating to geographical data, health status, health utilization, and chronic diseases, among others. Accordingly, the survey collected various health variables such as self-assessed health status and the use of periodic health check-ups, which can be used to assess the demand and equity in preventive healthcare. The survey used a two-stage sampling approach, with a total of 15,265 individuals covering the 13 regions of Saudi Arabia who were randomly selected for participation. The present analysis was limited to respondents who provided complete information on all variables of interest. Therefore, this analysis was based on a sample of 11,528 respondents.

### Measurement Variables

The FHS collected information on the health status of individuals and whether or not they undertake periodic health check-ups with the following question: “do you make any periodic check-ups to check your health?” Preventive check-ups include check-up for breast cancer, cervical cancer, colorectal cancer, prostate cancer, diabetes, blood pressure, cholesterol, teeth and mouth, and others. The time period for check-ups including any health check-ups that done weekly, monthly, every 3 months, every 6 months, annual, and more than 1 year. The response to this question was given a value of 1 for “yes” and 0 for “no.” This binary variable was then used as the dependent variable in examining the socioeconomic determinants and assessing inequalities in undertaking preventive health check-ups in the KSA.

Socioeconomic and demographic characteristics, including age, gender, marital status, education level, nationality, monthly income, health insurance coverage, health status, and region of residence, were used as independent variables, with income and education level serving as the socioeconomic status (SES) indicators among the respondents. The age variable was divided into five categories: 18 to 29 (the reference category), 30 to 39, 40 to 49, 50 to 59, and ≥60 years. Gender was assigned a value of 1 if the respondent is male and 0 if female. Marital status was also captured as a binary variable, with a value of 1 given for married respondents and 0 otherwise (including never married, divorced, and widowed). Nationality was assigned a value of 1 if the respondent is Saudi and 0 if non-Saudi. Health insurance was also captured as a binary variable with 1, assigned if the respondent is covered by health insurance and 0 otherwise. Health status was grouped into five categories: very bad (those who perceive themselves to be in very bad health status; reference group), bad, mediocre, good, and very good. Education level was grouped into the following categories: below primary school (reference), primary school, intermediate school, high school, and higher education. Monthly income [Saudi Riyal (SR); 1 SR = USD 0.27] was grouped into eight categories: less than SR 3,000 (reference category), SR 3,000 to <5,000, SR 5,000 to <7,000, SR 7,000 to <10,000, SR 10,000 to <15,000, SR 15,000 to <20,000, SR 20,000 to <30,000 and SR 30,000 or more. To account for regional differences, the region variable was grouped into the 13 administrative regions: Riyadh (reference), Albaha, Aljouf, Aseer, Eastern Region, Haiel, Jazan, Madenah, Mekkah, Najran, Northern Borders, Qaseem, and Tabouk.

### Data Analyses

The analysis for this study was conducted in various steps. Firstly, univariate analysis was performed using percentages and frequencies of the respondents' characteristics. Secondly, bivariate analysis was employed where cross-tabulation of the dependent variables and the associated frequencies were compared using the Chi-squared test. Thirdly, multivariate logistic regression models were estimated to examine the association between the dependent variable and the socio-economic factors (income, education, age, gender, marital status, and region). Additionally, the methodology of (24) was adopted to measure socio-economic inequalities in undertaking preventive health check-ups. All analyses were carried out using Stata/MP version 15.1.

Inequality analysis was performed using a bivariate index methodology initially proposed by Wagstaff et al. (24), known as the concentration index, which quantifies the degree of socioeconomic-related inequality in health or healthcare use. The concentration index is derived from the concentration curve, which is a visual representation that plots the cumulative percentage of the healthcare utilization variable on the vertical axis against the cumulative share of the population (ranked from the lowest to the highest by an indicator of SES) on the horizontal axis. A concentration curve above (below) the line of equality indicates greater use of healthcare services among the poor (rich) (25). The further the concentration curve is away from the line of equality (i.e., the 45-degree line), the greater the degree of inequality (26).

The concentration index can be either negative or positive, which ranges between +1 and −1. A negative concentration index means that access to preventive healthcare is concentrated on individuals with relatively low income, whereas a positive concentration index means that preventive healthcare is concentrated among the relatively rich. A concentration index of 0 means that no income-related inequality exists in preventive healthcare. Given that the variable used for assessing healthcare-seeking behavior is binary, the bounds of the concentration index may go beyond −1 or +1. Given this possibility, we used the Erreygers concentration index (27), which is a modified version of the concentration index that takes into account the binary nature of the dependent variable, expressed as:


(1) $$\begin{array}{r} ECI=8\;cov\left(h_{i},\;r_{i}\right) \end{array}$$

Where *ECI* is the Erreygers-corrected concentration index, *h*_*i*_ is the access to healthcare check-ups, *and r*_*i*_ is the individual relative rank in the wealth distribution.

### Decomposition of the Concentration Index: Explaining Socioeconomic-Related Inequality

To understand the contribution of each factor to the observed socioeconomic inequality, we decomposed the concentration index in terms of SES. This is an important analysis for policymakers to pinpoint the variables they will prioritize to reduce the observed socioeconomic inequality. We employed the approach proposed by Wagstaff et al. (28) to partition inequality into its contributing factors. To understand the decomposition concept, assuming that *Y*_*i*_ utilization of preventive healthcare services is a linear and additively separable function *X*_*j*_, the vector of covariates is obtained as:


(2) $$\begin{array}{r} Y_{i}=\;\alpha +\beta_{j}X_{ji}+\varepsilon_{i} \end{array}$$

The concentration index can then be expressed as a weighted sum of the aggregated indices of the different explanatory variables in the model for preventive healthcare-seeking behavior with respect to the measure of SES (28), as follows:


(3) $$\begin{array}{r} CI=\overset{J}{\underset{j=1}{\sum }}\beta_{j}\frac{{\bar{X}}_{j}}{\mu }CI_{j}+\;\frac{GCI_{\varepsilon }}{\mu } \end{array}$$

Where β_*j*_ represents the partial effect of healthcare determinants, *CI*_*j*_ represents the concentration indices of ${\bar{X}}_{j}$, and *GCI*_ε_ is the generalized concentration index of the error term. Equation 3 illustrates that the contribution of each variable to inequality is based on the interaction between the elasticity of healthcare use ($\beta_{j}*\frac{{\bar{X}}_{j}}{\mu }$) with respect to that variable and SES-related inequality in the distribution of the variable.

Following Van de Poel et al. (29), we can then decompose the above expression into the final equation as follows:


(4) $$\begin{array}{r} EI=4\left[\overset{J}{\underset{j=1}{\sum }}\beta_{j}*{\bar{X}}_{j}*CI_{j}+GCI_{\varepsilon }\right] \end{array}$$

### Ethical Clearance

This study was based on the use of secondary data from the FHS, which was conducted, commissioned, funded, and managed in 2018 by GaStat, who were in charge of all ethical procedures. All procedures performed in this study involving human participants complied with institutional and/or national research committee ethical standards, and with the 1964 Helsinki Declaration and subsequent amendments or equivalent ethical standards. Informed consent was obtained from all participants. All personal identifiers were removed from the dataset by GaStat to allow for secondary data use. GaStat granted permission to use the data, and thus, no further clearance was necessary as this was performed at the data collection phase.

## Results

Table 1 shows the socioeconomic characteristics of the respondents. Almost 50% of the respondents reported undertaking periodic preventive health check-ups over the study period. A majority (32.4%) of the respondents were aged below 29 years, whereas approximately 18% of the respondents were aged 60 years and above. Most of the respondents were male (54.3%). A large proportion (33.6%) had secondary school education, and the lowest proportion reported a primary level education. In terms of nationality, the majority of respondents were Saudi nationals, making up 75% of the sample. Approximately 32% of the sample was covered by health insurance. About two-thirds (64%) of the sample were married and about 10% earned below 3,000 SR.

**Table 1:** Socioeconomic characteristics of the respondents (*N* = 11,528).

**Variables**	**Frequency**	**%**
Periodic health check-ups	5,680	49.27
**Age**		
18–29	3,733	32.38
30–39	2,398	20.80
40–49	1,805	15.66
50–59	1,569	13.61
≥60	2,023	17.55
**Gender**		
Female	5,270	45.71
Male	6,258	54.29
**Marital status**		
Unmarried	4,133	35.85
Married	7,395	64.15
**Education level**		
Below primary school	2,227	19.32
Primary school	1,246	10.81
Intermediate school	1,893	16.42
Secondary school	3,870	33.57
Higher education	2,292	19.88
**Nationality**		
Non-Saudi	2,882	25.00
Saudi	8,646	75.00
**Monthly income (Saudi Riyal)**		
<3,000	1,062	9.21
3,000 to <5,000	1,851	16.06
5,000 to <7,000	1,777	15.41
7,000 to <10,000	2,220	19.26
10,000 to <15,000	2,174	18.86
15,000 to <20,000	1,118	9.70
20,000 to <30,000	721	6.25
≥30,000	605	5.25
**Health insurance**		
Uninsured	7,880	68.36
Insured	3,648	31.64
**Health status**		
Very bad	121	1.05
Bad	374	3.25
Mediocre	1,176	10.20
Good	2,368	20.54
Very good	7,489	64.96
**Region**		
Riyadh	1,652	14.33
Albaha	745	6.46
Aljoof	386	3.35
Aseer	582	5.05
Eastern Region	1,049	9.10
Haiel	745	6.46
Jazan	663	5.75
Madenah	834	7.23
Mekkah	2,003	17.38
Najran	408	3.54
Northern Border	411	3.57
Qassim	1,261	10.94
Tabuk	789	6.84

**Figure 1:**
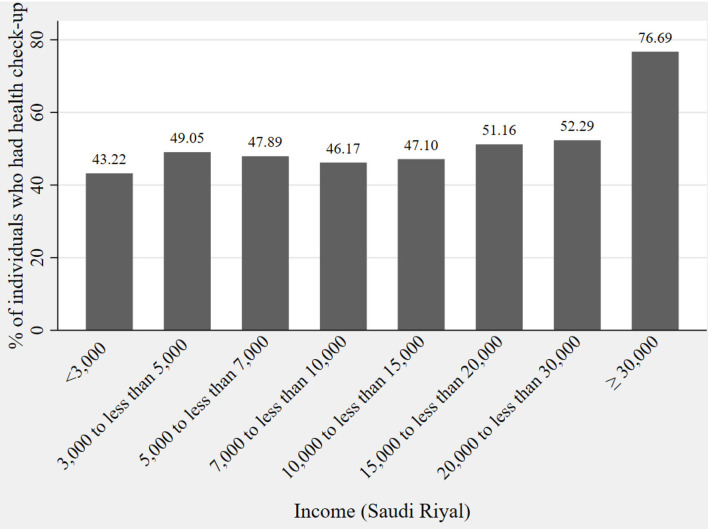
Distribution of periodic preventive health check-ups across income.

Considering the distribution of socioeconomic characteristics, we performed a visual inspection of the income-related inequality in preventive healthcare-seeking behavior. Figure 1 shows that the lowest percentage of people who undertook periodic preventive health check-ups was in the lowest income category. A high number of respondents in the highest income category (≥30,000 SR) reported undertaking periodic preventive health check-ups. This relation indicated the existence of income-related inequality in periodic preventive health check-ups.

Bivariate analysis was performed to assess the link between the socioeconomic factors and whether or not a respondent undertook preventive health check-ups. The results are presented in Table 2. In the table, we see that there appears to be a significant association between age and undertaking of check-ups and this increases with age. The percentage of males who undertook check-ups was higher than that of females. To avoid verbosity in the table, see Table 2.

**Table 2:** Bivariate analysis of the relationship between undertaking preventive health check-ups across the respondents' characteristics (*N* = 11,528).

**Variable**	**No check-ups**		**Check-ups**		**Total**	**Chi-square**	***P*-value**
	**No**.	**%**	**No**.	**%**			
**Age**							
18–20	2,789	75	944	25	3,733		
30–39	1,448	60	950	40	2,398		
40–49	856	47	949	53	1,805	2,292.51	<0.001
50–59	452	29	1,117	71	1,569		
≥60	303	15	1,720	85	2,023		
**Gender**							
Female	2,505	48	2,765	53	5,270		
Male	3,343	53	2,915	47	6,258	39.66	<0.001
**Marital status**							
Unmarried	2,730	66	1,403	34	4,133		
Married	3,118	42	4,277	58	7,395	605.39	<0.001
**Education level**							
Below primary school	702	32	1,525	69	2,227		
Primary school	549	44	697	56	1,246		
Intermediate school	1,035	55	858	45	1,893	517.29	<0.001
Secondary school	2,343	61	1,527	40	3,870		
Higher education	1,219	53	1,073	47	2,292		
**Nationality**							
Non-Saudi	1,542	54	1,340	46	2,882	11.85	<0.001
Saudi	4,306	50	4,340	50	8,646		
**Monthly income (SR)**							
<3,000	603	57	459	43	1,062		
3,000 to <5,000	943	51	908	49	1,851		
5,000 to <7,000	926	52	851	48	1,777		
7,000 to <10,000	1,195	54	1,025	46	2,220	215.83	<0.001
10,000 to <15,000	1,150	53	1,024	47	2,174		
15,000 to <20,000	546	49	572	51	1,118		
20,000 to <30,000	344	48	377	52	721		
≥30,000	141	23	464	77	605		
**Health insurance**							
Uninsured	4,294	55	3,586	46	7,880	141.13	<0.001
Insured	1,554	43	2,094	57	3,648		
**Health status**							
Very bad	4	3	117	97	121		
Bad	19	5	355	95	374		
Mediocre	110	9	1,066	91	1,176		
Good	707	30	1,661	70	2,368	2,419.63	<0.001
Very good	5,008	67	2,481	33	7,489		
**Region**							
Riyadh	944	57	708	43	1,652		
Albaha	289	39	456	61	745		
Aljoof	240	62	146	38	386		
Aseer	355	61	227	39	582		
Eastern Region	383	37	666	64	1,049		
Haiel	228	31	517	69	745		
Jazan	415	63	248	37	663		
Madenah	551	66	283	34	834	629.22	<0.001
Mekkah	1,102	55	901	45	2,003		
Najran	291	71	117	29	408		
Northern border	193	47	218	53	411		
Qassim	456	36	805	64	1,261		
Tabuk	401	51	388	49	789		
**Total**	**5,848**	**51**	**5,680**	**49**	**11,528**		

We next assessed the determinants of undertaking periodic preventive health check-ups (Table 3). Men were less likely to undertake periodic preventive health check-ups [odds ratio (OR) = 0.66; 95% confidence interval (CI) = 0.60–0.73; *P* ≤ 0.01] than women. There also appeared to be a strong association between periodic preventive health check-ups and age. Respondents with a higher education level were more likely to undertake periodic preventive health check-ups (OR = 1.26; 95% CI = 1.06–1.50; *P* ≤ 0.01) compared to those with an education below primary school level. In addition, respondents who assessed themselves to be in very good health, across all the health status categories, were less likely to undertake periodic preventive health check-ups (OR = 0.02; 95% CI = 0.01–0.06; *P* ≤ 0.01) compared with those who assessed themselves to be in very bad health status. There was no significant association between undertaking periodic preventive health check-ups and nationality. The likelihood of undertaking periodic preventive health check-ups was higher for those who were covered by health insurance than for those not covered by any form of health insurance (OR = 2.29; 95% CI = 2.02–2.59; *P* ≤ 0.01).

**Table 3:** Association between undertaking periodic preventive health check-ups and socioeconomic factors (logistic regression).

**Variables**	**Odds ratio**	**95% Confidence interval**
**Age**		
18–29	Ref	
30–39	1.40^***^	(1.21–1.61)
40–49	1.83^***^	(1.56–2.14)
50–59	3.04^***^	(2.55–3.64)
≥60	4.86^***^	(3.93–6.00)
**Gender**		
Female	Ref	
Male	0.66^***^	(0.60–0.73)
**Marital status**		
Unmarried	Ref	
Married	1.64^***^	(1.45–1.85)
**Education level**		
Below primary school	Ref	
Primary school	1.05	(0.87–1.26)
Intermediate school	1.24^**^	(1.05–1.48)
Secondary school	1.16^*^	(0.98–1.36)
Higher Education	1.26^***^	(1.06–1.50)
**Nationality**		
Non-Saudi	Ref	
Saudi	1.06	(0.92–1.22)
**Monthly income (Saudi Riyal)**		
<3,000	Ref	
3,000 to <5,000	1.37^***^	(1.14–1.66)
5,000 to <7,000	1.33^***^	(1.10–1.61)
7,000 to <10,000	1.09	(0.90–1.32)
10,000 to <15,000	1.33^***^	(1.09–1.62)
15,000 to <20,000	1.69^***^	(1.35–2.11)
20,000 to <30,000	1.70^***^	(1.32–2.18)
≥30,000	4.00^***^	(3.01–5.32)
**Health insurance**		
Uninsured	Ref	
Insured	2.29^***^	(2.02–2.59)
**Health status**		
Very bad	Ref	
Bad	0.41	(0.12–1.36)
Mediocre	0.26^**^	(0.09–0.80)
Good	0.06^***^	(0.02–0.19)
Very good	0.02^***^	(0.01–0.06)
**Region**		
Riyadh	Ref	
Albaha	2.67^***^	(2.13–3.36)
Aljoof	1.02	(0.76–1.36)
Aseer	0.87	(0.69–1.09)
Eastern Region	1.92^***^	(1.59–2.32)
Haiel	4.66^***^	(3.78–5.73)
Jazan	1.26^**^	(1.01–1.58)
Madenah	0.65^***^	(0.53–0.80)
Mekkah	1.17^*^	(1.00–1.37)
Najran	0.73^**^	(0.55–0.96)
Northern Border	2.52^***^	(1.96–3.24)
Qassim	2.36^***^	(1.97–2.84)
Tabuk	1.23^*^	(0.99–1.52)
Constant	4.61^***^	(1.48–14.33)
Observations	11,528	
Pseudo R2	0.273	
Log Likelihood	−5,811	
Wald Chi	2,697	
Pro>Chi2	0.000	

*** *P < 0.01;*

** *P < 0.05;*

* *P < 0.1*.

**Figure 2:**
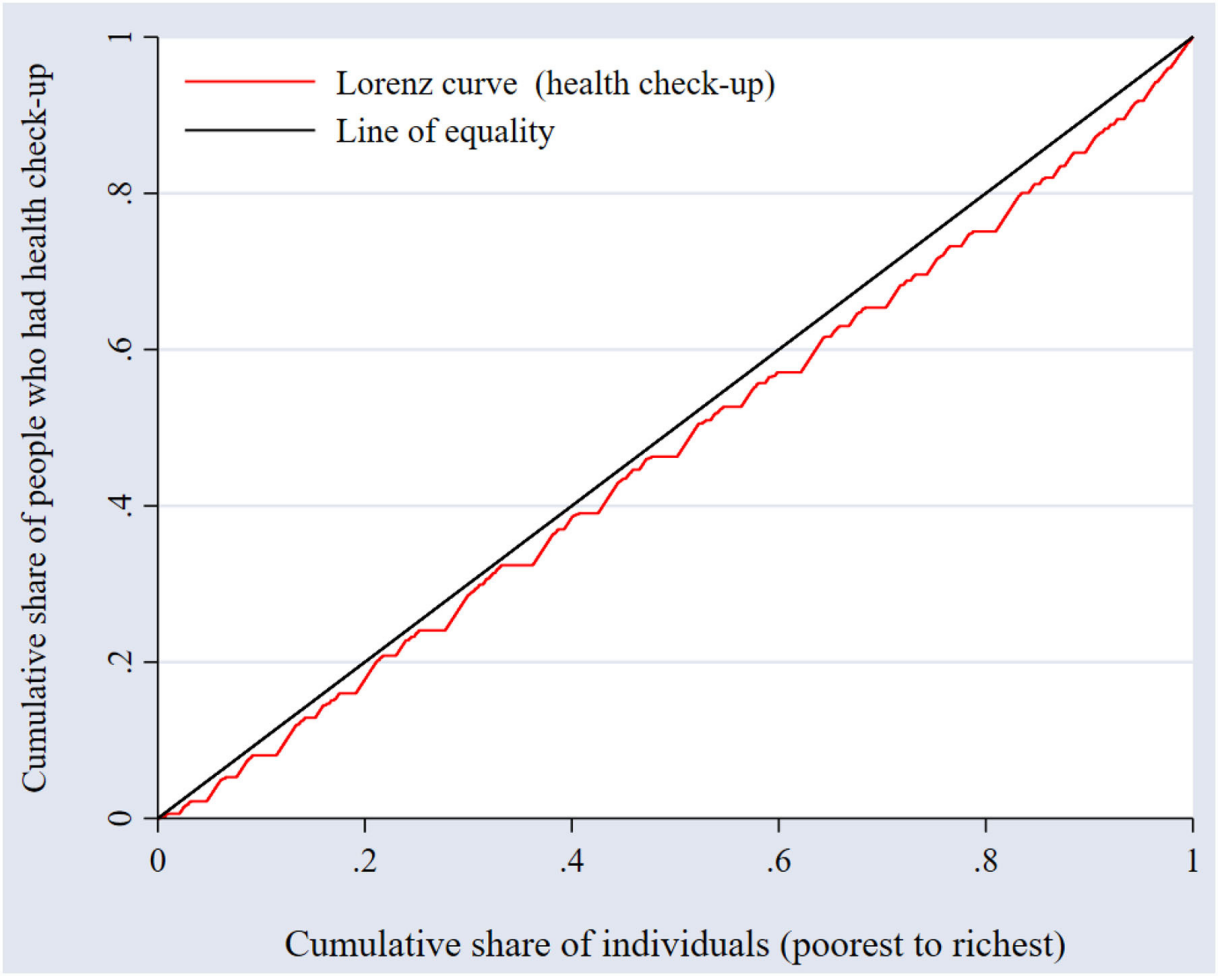
Income-related inequality in undertaking periodic preventive health check-ups.

We further assessed the wealth-related inequality in undertaking periodic preventive health check-ups. Visual inspection of the concentration curves was first performed, as shown in Figure 2.

In Figure 2, the concentration curve lies to the right of the line of equality, indicating that there is inequality in undertaking periodic preventive health check-ups against the poor. In other words, undertaking periodic preventive health check-ups is concentrated among individuals with higher income as opposed to individuals with lower income. Because the concentration index does not indicate the magnitude of inequality, we calculated the concentration index for preventive health check-ups, which was 0.0831 (95% confidence interval: 0.062–0.104, *P* < 0.001), representing overall inequality, thereby supporting the findings from the concentration curves.

Apart from the overall inequality assessment, Table 4 shows the degree of inequalities by region, which was calculated to assess if there is heterogeneity in inequality across regions. The justification for this analysis is the fact that various regions in the KSA may have different conditions that could influence the socioeconomic inequalities in the uptake of preventive health check-ups.

**Table 4:** Erreygers indices of inequality in preventive health check-ups by region.

**Region**	**Erreygers Index**	**Confidence Interval**	**N**
Riyadh	0.034^**^	[0.002, 0.066]	1,652
Albaha	0.012	[−0.021, 0.045]	745
Aljoof	−0.008	[−0.082, 0.066]	386
Aseer	0.026	[−0.032, 0.085]	582
Eastern Region	0.013	[−0.013, 0.038]	1,049
Haiel	−0.036^***^	[−0.061, −0.010]	745
Jazan	0.049	[−0.009, 0.107]	663
Madenah	−0.049^*^	[−0.102, 0.004]	834
Mekkah	0.023^*^	[−0.004, 0.051]	2,003
Najran	0.086^*^	[−0.004, 0.177]	408
Northern Border	−0.113^***^	[−0.165, −0.061]	411
Qassim	0.142^***^	[0.121, 0.162]	1,261
Tabuk	−0.089^***^	[−0.130, −0.048]	789

* *P < 0.10;*

** *P < 0.05;*

*** *P < 0.01*.

Some important variations across the various regions of the KSA were observed. On the one hand, the Erreygers indices were negative for Haiel, Madenah, Northern Border, and Tabuk, suggesting that the poor in these regions undertake more preventive health check-ups. On the other hand, the Erreygers indices were positive for Riyadh, Mekkah, Najran, and Qassim, suggesting higher uptake of preventive health check-ups among the relatively rich. In some districts, the Erreygers indices were not statistically significant, including Albaha, Aljoof, and Aseer. These varied results obtained across the regions thus confirmed heterogeneity.

Finally, we analyzed how specific factors contribute to the observed inequality (Table 5). The analysis showed that income contribute to an aggregate of nearly 24.4%, followed by regions at 23.4% and education at 17.8%. By contrast, having health insurance appears to have an effect on reducing inequality. It should be noted that the value of the residual is a bit large, and that the decomposition which has been done in this explains only about 60% of the overall inequality. This may suggest the existence of other factors contribute to inequality that are not included in the analysis.

**Table 5:** Decomposition of the Erreygers index according to demographic variables.

**Variables**	**B**	**Concentration Index**	**Elasticity**	**Contribution**	**%**
**Age**					
18–29					
30–39	0.064	−0.033	0.027	−0.002	−2.1
40–49	0.136	0.011	0.043	0.001	1.2
50–59	0.236	0.017	0.065	0.002	2.6
≥60	0.294	−0.008	0.105	−0.002	−2.1
**Gender**					
Female					
Male	−0.073	−0.045	−0.080	0.007	8.6
**Marital status**					
Unmarried					
Married	0.076	0.002	0.099	0.000	0.5
**Education level**					
Below primary school					
Primary school	0.012	−0.146	0.003	−0.001	−0.9
Intermediate school	0.042	−0.033	0.014	−0.001	−1.1
Secondary school	0.034	0.069	0.023	0.003	3.8
Higher Education	0.064	0.261	0.026	0.013	16.0
**Nationality**					
Non-Saudi					
Saudi	0.020	0.110	0.030	0.007	7.8
**Monthly income (Saudi Riyal)**					
?3,000					
3,000 to <5,000	0.155	−0.034	0.020	−0.001	−1.6
5,000 to <7,000	−0.012	−0.257	−0.001	0.000	0.5
7,000 to <10,000	−0.023	0.120	−0.002	−0.001	−0.7
10,000 to <15,000	0.114	0.136	0.021	0.006	6.7
15,000 to <20,000	0.286	0.136	0.038	0.010	12.1
20,000 to <30,000	0.023	−0.039	0.003	0.000	−0.2
≥30,000	−0.075	−0.293	−0.011	0.006	7.6
**Health insurance**					
Uninsured					
Insured	0.141	−0.050	0.090	−0.009	−10.7
**Health status**					
Very bad					
Bad	−0.042	−0.166	−0.003	0.001	1.1
Mediocre	−0.043	−0.061	−0.009	0.001	1.3
Good	−0.222	0.017	−0.093	−0.003	−3.6
Very good	−0.453	0.009	−0.598	−0.010	−12.3
**Region**					
Riyadh					
Albaha	0.023	−0.157	0.008	−0.002	−2.9
Aljoof	−0.053	−0.014	−0.004	0.000	0.1
Aseer	0.177	−0.252	0.013	−0.006	−7.6
Eastern Region	0.165	0.330	0.037	0.024	28.7
Haiel	0.032	−0.119	0.004	−0.001	−1.2
Jazan	−0.069	−0.045	−0.076	0.007	8.1
Madenah	0.067	−0.033	0.028	−0.002	−2.2
Mekkah	0.132	0.011	0.042	0.001	1.1
Najran	0.227	0.017	0.063	0.002	2.5
Northern Border	0.282	−0.008	0.100	−0.002	−2.0
Qassim	0.006	−0.146	0.001	0.000	−0.5
Tabuk	0.029	−0.033	0.010	−0.001	−0.7
Explained inequality				0.047	57.9
Overall inequality				0.083	
Residual				0.036	

## Discussion

This study examined the extent of socioeconomic inequalities in undertaking health check-ups in the KSA. Furthermore, the observed socioeconomic inequality in undertaking health check-ups was decomposed to identify the main determinates contribute to the observed inequality. The results revealed that nearly half of the respondents indicated that they undertook some preventive health check-ups. This proportion is comparatively higher than that reported in most regions, but is lower according to most European standards and that reported in the United States (30). Such uptake may signal a good system that can help in fighting against chronic diseases, which could be detected much earlier with preventive healthcare. Although our findings suggest relatively high uptake, closer examination of the inequality dimension suggested other bottlenecks that put the equity of the system into question.

Along the gender dimension, our findings suggest that men are less likely to undertake periodic preventive health check-ups than women. This finding supports previous reports in the context of the United States, Austria, and the United Kingdom (31, 32). The gender difference in the uptake of preventive services may arise as a result of biological requirements, since most women may be forced to undergo some health checks during prenatal and postnatal care at various phases of their lives.

Despite observing uptake of health check-ups by close to half the population of the KSA, the situation appears to be less promising in light of the inequality analysis. Indeed, the concentration index as well as the concentration curves suggest that much of this uptake is actually concentrated among those with higher incomes. A positive concentration index indicates that the poor are at a disadvantage in terms of the uptake of preventive healthcare services (27, 33). Indeed, this finding does not exist in a vacuum and does not appear to be unique to the situation in the KSA. In Germany, despite a different cultural context than that of the KSA, socioeconomically disadvantaged groups appear to be less likely to use preventive healthcare services than socioeconomically privileged groups (13, 34, 35). Although concentration indices were not calculated in these previous studies, the overall message found in the context of various healthcare systems outside of the KSA points to the fact that the disadvantaged are less likely to undertake preventive healthcare, even in situations where healthcare is free, and this disparity can be much more severe in some regions, including in Africa (36). Not only that, it also concurs with other studies established in EMRO countries, which also showed pro-rich uptake in dental care (37) though in contrast with uptake of public healthcare services (38). Underlying factors such as differences in incomes and geography likely contribute to the gap in the uptake of preventive healthcare.

Decomposition of the concentration index suggested that region, education, and income level are the greatest drivers of the inequality in the Erreygers index. These factors have also been identified as drivers of socioeconomic-related inequality in preventive health check-ups uptake in China (39) and India (40). One potential explanation might be that owing to the long waiting times at public healthcare facilities (41), those with better incomes may be in a position to support themselves to use private healthcare services or purchase health insurance, which has been found to contribute to easy access to the healthcare (18, 42). Additionally, those with higher education may be much more aware about the benefits of carrying out preventive health check-ups. However, there is no other study that performed a decomposition of the concentration index in the surrounding regions of Saudi Arabia, which makes it difficult to directly compare our results to previous findings.

Although important results have been established in the current study, we are also aware of the limitations in the study design and analysis. Firstly, our analysis was not designed to present casual relationships of the evidence, as this was beyond the scope of the study; thus, these results must be interpreted as indicating associations and not causality. Secondly, the results may also suffer from the problem of recall bias by some individuals regarding health check-ups. Recall bias is a common problem in most surveys in which the responses to questions rely on memory. Thirdly, our data does not use equivalence scales for adjustment of incomes, thus we do not have control household income adjustment.

## Conclusions

The aim of this study was to determine the presence of any socioeconomic inequalities in preventive health check-ups uptake in the KSA. The study was motivated by evidence that undertaking preventive healthcare is an important strategy in the fight against several diseases, but that various socioeconomic circumstances are attributed to the uptake of preventive health check-ups (43–46). We first performed a determinant analysis to identify the extent to which socioeconomic variables affect the uptake of preventive health check-ups. We then calculated concentration indices and constructed associated concentration curves to assess the socioeconomic inequality in preventive healthcare uptake. In the determinant analysis, the study found that being older adults, highly educated, insured, and married were associated with a greater likelihood of undertaking preventive health check-ups. Regarding socioeconomic inequality, preventive healthcare uptake appears to be concentrated among the wealthier individuals. Although the overall picture points to the fact that socioeconomic inequality favors the relatively rich, we also observed some differences in terms of socioeconomic inequality across regions. From a policy perspective, these results suggest that the government should improve the incomes of the less privileged to increase the uptake of preventive health check-ups for this group. Even though raising income could be seen as a solution, it can be said that a large number of developing, and even developed countries, are struggling to improve the economic status of the poor since decades. Therefore, the government of Saudi Arabia should develop intervention programs and strategies that promote the uptake of health check-ups among the vulnerable group to reduce inequalities. Of particular importance is the need for more health-related education programmes among the poor and those with lower education in order to raise their awareness on the benefits and advantages of conducting health examinations.

## Data Availability Statement

The datasets generated and/or analysed during the current study are not publicly available due to privacy, confidentiality, and other restrictions. Access to data can be gained through the General Authority for Statistics in Saudi Arabia.

## Ethics Statement

This study did not require ethical approval because we used secondary data. Furthermore, the data were de-identified prior to the analysis. The outcomes of the analysis do not allow for re-identification and the use of data cannot result in any damage or distress to the participants.

## Author Contributions

MKA and GCC: conceptualization, investigation, methodology, data curation, formal analysis, software, writing—original draft preparation, and writing—review and editing. Both authors contributed to the article and approved the submitted version.

## Funding

This research was funded by the Institutional Fund Projects under grant number (IFPIP-72-120-1442). The funders had no role in study design, data collection and analysis, decision to publish, or preparation of the manuscript.

## Conflict of Interest

The authors declare that the research was conducted in the absence of any commercial or financial relationships that could be construed as a potential conflict of interest.

## Publisher's Note

All claims expressed in this article are solely those of the authors and do not necessarily represent those of their affiliated organizations, or those of the publisher, the editors and the reviewers. Any product that may be evaluated in this article, or claim that may be made by its manufacturer, is not guaranteed or endorsed by the publisher.
